# Risk of total hip arthroplasty following lumbar fusion surgery in a nationwide cohort study

**DOI:** 10.1038/s41598-026-35894-8

**Published:** 2026-01-18

**Authors:** Youngoh Bae, Seung Won Lee, Seondeok Seo, Jongseung Han, Daewon Yang, Jin Hoon Park, Hohyun Jung

**Affiliations:** 1 Department of Neurological Surgery, Asan Medical Center, University of Ulsan College of Medicine, Seoul, 05505 Republic of Korea; 2 Department of Precision Medicine, Sungkyunkwan University School of Medicine, Suwon, 16419 Republic of Korea; 3 Personalized Cancer Immunotherapy Research Center, Sungkyunkwan University School of Medicine, Suwon, 16419 Republic of Korea; 4 Department of Artificial Intelligence, Sungkyunkwan University, Suwon, 16419 Republic of Korea; 5 Department of Family Medicine, Kangbuk Samsung Hospital, Sungkyunkwan University School of Medicine, Seoul, 03181 Republic of Korea; 6 Department of Information and Statistics, Chungnam National University, Daejeon, 34134 Republic of Korea; 7 Department of Statistics, Sungshin Women’s University, Seoul, 02844 Republic of Korea; 8 Center for Data Science, Sungshin Women’s University, Seoul, 02844 Republic of Korea; 9 Human-Centered AI Institute, Sungshin Women’s University, Seoul, 02844 Republic of Korea

**Keywords:** Lumbar fusion surgery, Total hip arthroplasty, Joint degeneration, Cohort study, Diseases, Health care, Medical research, Rheumatology, Risk factors

## Abstract

Alterations in spinopelvic alignment following lumbar fusion surgery (LFS) have been suggested to influence hip biomechanics, yet long-term population-based evidence regarding the risk of total hip arthroplasty (THA) remains limited. Using a nationwide cohort from the Korean National Health Insurance Service, we conducted a retrospective study applying a 3-year washout period and up to 10 years of follow-up. Patients who underwent LFS with deformity correction or sacropelvic fixation were propensity score–matched to individuals without LFS. The primary outcome was incident THA, while total knee arthroplasty (TKA) was evaluated as a comparison outcome. Cox proportional hazards models were used to estimate adjusted hazard ratios, with subgroup analyses performed according to age, sex, body mass index, and income. Patients undergoing LFS showed a significantly increased risk of THA compared with matched controls (adjusted hazard ratio 2.26; 95% confidence interval 1.33–3.82), with greater risk observed among older adults, women, individuals with higher body mass index, and those with higher income. In contrast, the association between LFS and TKA was weaker and not statistically significant. These findings suggest that the hip joint may be particularly vulnerable to biomechanical changes following lumbar fusion, highlighting the importance of long-term monitoring of hip health in patients undergoing LFS.

**Supplementary Information:**

The online version contains supplementary material available at 10.1038/s41598-026-35894-8.

## Introduction

Degenerative lumbar diseases are a common cause of functional impairment in the elderly population, and with population aging and advances in surgical techniques, the utilization of lumbar fusion surgery (LFS) has continued to increase^1,2^. Although LFS effectively stabilizes the lumbar spine affected by instability or degenerative changes, it can alter the biomechanical interactions among the spine, pelvis, and hip, which are closely related to sagittal alignment^3,4^ .

The spine, pelvis, and hip function as an interdependent kinetic chain to maintain sagittal balance^5^. In particular, posterior pelvic tilt serves as an important compensatory mechanism by regulating acetabular orientation and redistributing mechanical load across the hip joint during sitting and ambulation^6^. However, LFS, especially when extended to the sacrum or pelvis, restricts pelvic rotation, thereby forcing the hip joint to absorb excessive flexion–extension stress. This altered biomechanical demand may accelerate adjacent hip degeneration and subsequently increase the risk of total hip arthroplasty (THA)^1–5^.

Motion analysis and finite element modeling studies have reported that sacral-pelvic fixation increases hip joint loading by eliminating compensatory pelvic motion^1–3^. In addition, increased hip joint stress and angular motion have been observed in cases involving iliac screw fixation^4,5^, and these biomechanical changes have been proposed as a key mechanism underlying hip degeneration following spinal fusion.

Furthermore, clinical studies have reported that a greater preoperative mobility of the sacroiliac joint (SIJ) or a postoperative decrease in pelvic incidence is associated with an increased risk of progression of hip osteoarthritis^6,7^.

In contrast, some studies have reported that lumbar fusion performed without sacral or pelvic fixation may not independently increase the risk of THA^8^, indicating that clinical uncertainty regarding THA risk after LFS remains. The impact of LFS on the hip joint is likely heterogeneous, depending on the extent of fusion and individual patient characteristics. Nevertheless, prior studies have been limited by single-center designs, small sample sizes, short follow-up durations, or a lack of consideration of preoperative hip status, thereby constraining a precise causal interpretation of THA occurrence following LFS.

Previous studies, limited by small single-center cohorts, short follow-up, or inadequate assessment of preoperative hip status, have not established whether LFS increases long-term THA risk. Consequently, the association between LFS and THA remains unclear. To address these limitations, we conducted a large-scale, population-based study using the NHIS cohort with over 10 years of follow-up, applying a washout period and matched analyses. This study aimed to assess the long-term association between LFS and THA risk and to identify vulnerable subgroups relevant to surgical planning and postoperative care.

## Methods

### Data sources

The primary objective of this study was to evaluate the risk of THA by comparing patients who underwent LFS with matched control subjects. To complement the interpretation of THA as a single outcome, the occurrence of TKA was additionally analyzed as a secondary outcome. A retrospective cohort study was conducted using data from the National Health Insurance Service–National Sample Cohort (NHIS-NSC) and the National Health Screening Program (NHSP).

The study used data from 2002 to 2013 drawn from a 2.2% stratified random sample of Korean National Health Insurance beneficiaries, categorized into 1,476 strata by sex, age, and income. The single-payer National Health Insurance Service provides a comprehensive claims-based database, including ICD-10 diagnoses, procedures, prescriptions, sociodemographic data, and National Health Screening Program records, allowing reliable estimation of disease incidence^9^.

**Fig. 1:**
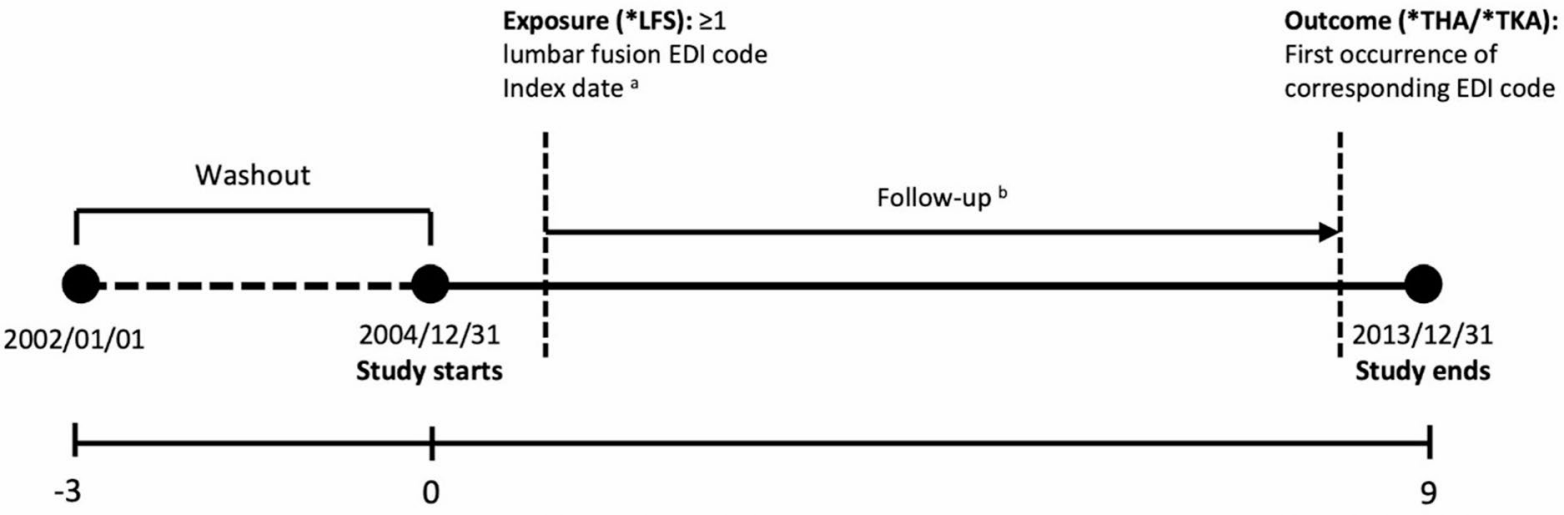
Study design and definition of baseline and follow-up (**a**) Index date: Defined as the date on which a patient received the first Electronic Data Interchange (EDI) procedure code corresponding to lumbar fusion surgery, indicating the initiation of exposure to LFS. (**b**) Follow-up: The period starting from the index date and continuing until the earliest occurrence of an outcome, death, or the end of the study period.

This study was exempted from review by the Institutional Review Board of Sungshin Women’s University (SSWUIRB-2025–055), and informed consent was waived because the data used were fully anonymized and could not be used to identify individual patients (Fig. 1).

### Case and control group selection

**Fig. 2:**
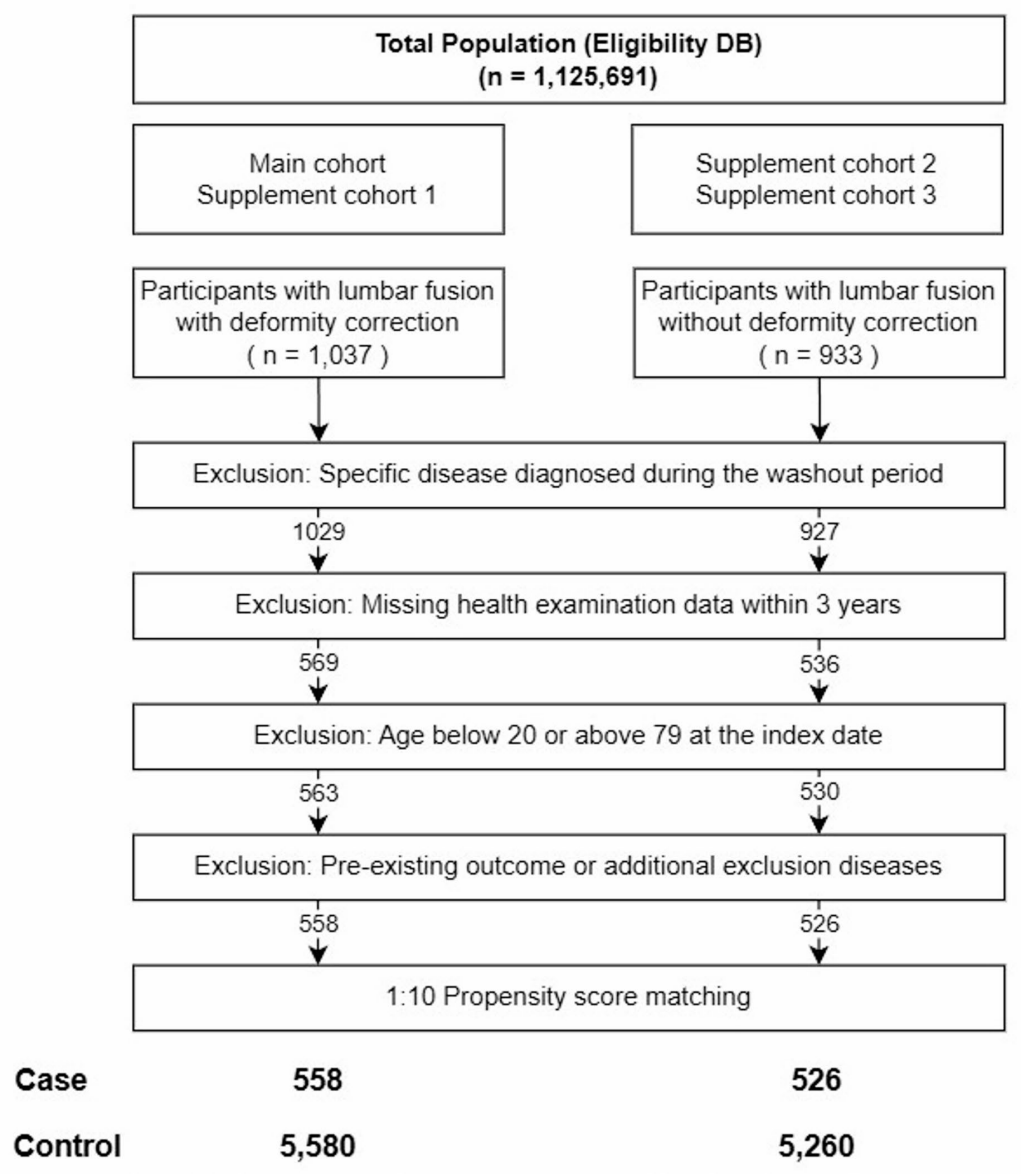
Flowchart for case and control group selection. THA, total hip arthroplasty; TKA, total knee arthroplasty.

Figure 2 illustrates the overall process of case and control group selection. The case group was defined as patients who had at least one recorded EDI procedure code for LFS during the observation period (2005–2013). Among these patients, those who underwent fusion procedures that included deformity correction or sacral/pelvic fixation were selected as the primary analysis cohort for evaluating the risk of THA.

In addition, supplementary analyses were conducted as follows: first, a cohort analysis using TKA as the outcome variable (Supplementary Cohort 1); second, among patients who underwent LFS without deformity correction or sacral/pelvic fixation, the risks of THA (Supplementary Cohort 2) and TKA (Supplementary Cohort 3) were evaluated. Detailed definitions and inclusion criteria are presented in Table 1. The detailed exclusion criteria and corresponding ICD-10/KCD codes are summarized in Supplementary Table S1.

**Table 1:** Electronic Data Interchange (EDI) procedure codes for exposure and outcome surgeries.

Disease	EDI code
Main cohort	
Exposure surgery	
LFS group with lumbar deformity correction or sacropelvic fixation	N0460 N0461 N0462 N0463 N0464 N0465 N0466 N0467 N0444 N0445 N0446 N0447
Outcome surgery	
Total Hip Arthroplasty, THA	N0711, N2070, N0715, N2710
Washout criteria	
Specific lumbar surgery	N0460, N0461, N0462, N0463, N0464, N0465, N0466, N0467, N0444, N0445, N0446, N0447
**Supplementary Cohort 1**	
Exposure surgery	
LFS group with lumbar deformity correction or sacropelvic fixation	N0460 N0461 N0462 N0463 N0464 N0465 N0466 N0467 N0444 N0445 N0446 N0447
Outcome surgery	
Total Knee Arthroplasty, TKA	N2072, N2077, N2712, N2717
**Supplementary Cohort 2**	
Exposure surgery	
LFS group without lumbar deformity correction or sacropelvic fixation	N0460 N0461 N0462 N0463 N0464 N0465 N0466 N0467
Outcome surgery	
Total Hip Arthroplasty, THA	N0711, N2070, N0715, N2710
**Supplementary Cohort 3**	
Exposure surgery	
LFS group without lumbar deformity correction or sacropelvic fixation	N0460 N0461 N0462 N0463 N0464 N0465 N0466 N0467
Outcome surgery	
Total Knee Arthroplasty, TKA	N2072, N2077, N2712, N2717

The index date for each case was defined as the date of the first LFS procedure. To establish an incident cohort and minimize reverse causality, an initial 3-year washout period (2002–2004) was applied, followed by a 9-year observation period^10^. Individuals with LFS during the washout period were excluded.

Participants were excluded if they had no health screening records within 3 years prior to the index date or were younger than 20 years or older than 80 years at the index date. To minimize confounding from pre-existing conditions, individuals with a history of the outcome events (THA or TKA) or additional exclusion diagnoses (e.g., N0712, N2071; Table 1) within 3 years before the index date were also excluded. The control group was selected from health screening participants with no history of LFS during the study period, applying the same washout period and exclusion criteria as those used for the case group.

To address baseline imbalances between the case and control groups, 1:10 propensity score matching was performed based on sex, age, and the year of health screening^11^. When missing values were present in covariates, single imputation using predictive mean matching was applied to minimize data loss.

### Study outcome

The primary outcome was incident THA, defined by EDI procedure codes N0711, N2070, N0715, and N2710, with occurrence defined as at least one recorded procedure; the date of the first procedure was used as the outcome index date. TKA was analyzed as a secondary outcome using EDI codes N2072, N2077, N2712, and N2717 and was defined using the same criteria as THA. Detailed code definitions are provided in Table 1. Participants were followed until death, outcome occurrence, or the end of follow-up (December 31, 2013) (Fig. 1).

### Variables

The analyses included sex, age, smoking status, alcohol consumption, height, weight, body mass index (BMI), total cholesterol, systolic blood pressure, diastolic blood pressure, fasting glucose, and income level as covariates. All variables were extracted from the NHSP record closest to the index date. Height and weight were treated as continuous variables, whereas sex, age, smoking status, alcohol consumption, BMI, total cholesterol, systolic and diastolic blood pressure, fasting glucose, and income level were categorized and included in the analyses as categorical variables.

### Statistical analysis

Baseline differences between the case and control groups were assessed using standardized differences, with an SD value of less than 0.1 indicating no meaningful imbalance between groups. Continuous variables were compared using Student’s *t*-test, and categorical variables were compared using the chi-square test. The incidence of THA was calculated as crude incidence rates per 1,000 person-years, and crude incidence rate ratios were used for comparisons between groups. The same analytical approach was applied to TKA as a secondary analysis.

Cumulative incidence of the outcome during the follow-up period was estimated using Kaplan–Meier survival analysis, and differences between groups were assessed for statistical significance using the log-rank test. To evaluate the association between LFS and the occurrence of THA, Cox proportional hazards regression models were applied to estimate aHRs with 95% confidence intervals. The same regression approach was used to perform a secondary analysis for TKA. The models were adjusted for sex, age, smoking status, alcohol consumption, BMI, total cholesterol, and income level.

The proportional hazards assumption was assessed using scaled Schoenfeld residuals and was confirmed to be satisfied. All statistical analyses were performed using R software (version 4.4.1), and a *p* value of < 0.05 was considered statistically significant.

### Subgroup analysis

To assess whether the association between LFS and the occurrence of THA varied according to key characteristics, subgroup analyses were performed based on age, sex, smoking status, weekly alcohol consumption, total cholesterol, income level, and BMI. Within each subgroup, Cox proportional hazards regression models were applied, and the stratifying variable was excluded from the model covariates. All statistical significance was evaluated using two-sided tests. Subgroup analyses for TKA were conducted using the same analytical approach as secondary analyses.

## Results

### Baseline characteristics

A total of 558 patients who underwent LFS with deformity correction and 5,580 matched control subjects were included after 1:10 propensity score matching based on sex, age, and year of health screening. After matching, all covariates showed standardized differences < 0.10, indicating good baseline balance. The age distribution was identical between groups, with individuals aged 60–69 years comprising the largest proportion (31.5%), and women accounted for 57.4% in both groups. Smoking status and weekly alcohol consumption were comparable. Mean body weight (63.04 kg vs. 62.77 kg) and height (159.97 cm vs. 160.08 cm) were similar, with no difference in BMI distribution. Total cholesterol, blood pressure, fasting glucose, and income level were also comparable between groups (Table 2). Similarly, in the LFS group without deformity correction or pelvic fixation and their matched controls, all covariates were well balanced (Table S2).

**Table 2:** Baseline characteristics of patients with lumbar fusion surgery with deformity correction and matched controls.

		Case group (n = 558) (%)	Control group (n = 5,580 (%)	Standardized difference	*p*-value
Age (years)	20–29	8 (1.43%)	80 (1.43%)	0.0	1
	30–39	27 (4.84%)	270 (4.84%)		
	40–49	100 (17.92%)	1000 (17.92%)		
	50–59	161 (28.85%)	1610 (28.85%)		
	60–69	176 (31.54%)	1760 (31.54%)		
	≥ 70	86 (15.41%)	860 (15.41%)		
Sex	Male	238 (42.65%)	2380 (42.65%)	0.0	1
	Female	320 (57.35%)	3200 (57.35%)		
Smoking status	Yes	102 (18.28%)	948 (16.99%)	0.06	0.8
	No	382 (68.46%)	3932 (70.47%)		
	Ex-Smoking	36 (6.45%)	347 (6.22%)		
Frequency of alcohol consumption (per week)	0	355 (63.62%)	3602 (64.55%)	0.05	0.79
	1–2	141 (25.27%)	1371 (24.57%)		
	≥ 3	52 (9.32%)	479 (8.58%)		
Weight (kg, mean ± SD)		63.04 ± 10.40	62.77 ± 10.00	0.03	0.56
Height (cm, mean ± SD)		159.97 ± 8.95	160.08 ± 8.91	0.01	0.79
BMI (kg/m^2^)	< 18.5	9 (1.61%)	81 (1.45%)	0.04	0.82
	18.5 to < 25	318 (56.99%)	3227 (57.83%)		
	≥ 25	230 (41.22%)	2268 (40.65%)		
Total cholesterol (mg/dL)	< 200	296 (53.05%)	2959 (53.03%)	0.03	0.9
	≥ 200	262 (46.95%)	2619 (46.94%)		
Systolic Blood Pressure (mmHg)	< 120	163 (29.21%)	1631 (29.23%)	0.03	0.97
	120 to < 140	285 (51.08%)	2882 (51.65%)		
	≥ 140	110 (19.71%)	1066 (19.10%)		
Diastolic Blood Pressure (mmHg)	< 80	251 (44.98%)	2489 (44.61%)	0.04	0.91
	80 to < 90	220 (39.43%)	2268 (40.65%)		
	≥ 90	87 (15.59%)	822 (14.73%)		
FBS (mg/dL)	< 100	359 (64.34%)	3651 (65.43%)	0.04	0.94
	100 to < 126	145 (25.99%)	1411 (25.29%)		
	≥ 126	54 (9.68%)	517 (9.27%)		
Income	Low	160 (28.67%)	1575 (28.23%)	0.01	0.86
	High	398 (71.33%)	4005 (71.77%)		

*BMI* body mass index, *FBS* fasting blood sugar.

### Incidence of THA and TKA

In the LFS group with deformity correction and pelvic fixation, the crude incidence rate of THA was significantly higher than in controls (6.71 vs. 2.83 per 1,000 person-years; IRR, 2.37; 95% CI, 1.42–3.96) (Table 3). This difference was pronounced among patients aged ≥ 60 years (16.53 vs. 6.48 per 1,000 person-years; IRR, 2.55; 95% CI, 1.50–4.34), whereas no significant difference was observed in those < 60 years.

In sex analyses, the risk of THA was significantly increased among female patients (IRR, 2.39; 95% CI, 1.39–4.12), with the highest incidence observed in women aged ≥ 60 years (IRR, 2.57; 95% CI, 1.46–4.53). In contrast, although an increasing trend in THA risk was observed among male patients, the association did not reach statistical significance.

**Table 3:** Crude incidence rates and incidence rate ratios of THA following lumbar fusion surgery with deformity correction.

		Case cohort (n = 558)			Reference cohort (n = 5,580)			IRR (95% CI)
		Cases	Person-years	IR per 1000 person-years (95% CI)	Cases	Person-years	IR per 1000 person-years (95% CI)	
All		18	2684.04	6.71 (3.73–10.06)	78	27,582.67	2.83 (2.21–3.48)	2.37 (1.42–3.96)
Age (years)	< 60	1	1655.56	0.60 (0.00–1.81)	9	16,927.9	0.53 (0.24–0.89)	1.14 (0.14–8.97)
	≥ 60	17	1028.47	16.53 (8.75–25.28)	69	10,654.78	6.48 (4.97–8.07)	2.55 (1.50–4.34)
Sex	Male	2	1263.33	1.58 (0.00–3.96)	9	12,914.3	0.70 (0.31–1.16)	2.27 (0.49–10.51)
	Female	16	1420.7	11.26 (6.33–16.89)	69	14,668.38	4.70 (3.61–5.86)	2.39 (1.39–4.12)
Sex & Age (years)	Male, < 60	0	854.63	0.00 (0.00–0.00)	0	8634.09	0.00 (0.00–0.00)	nan (nan–nan)
	Male, ≥ 60	2	408.7	4.89 (0.00–12.23)	9	4280.21	2.10 (0.93–3.50)	2.33 (0.50–10.77)
	Female, < 60	1	800.93	1.25 (0.00–3.75)	9	8293.81	1.09 (0.48–1.81)	1.15 (0.15–9.08)
	Female, ≥ 60	15	619.77	24.20 (12.91–37.11)	60	6374.57	9.41 (7.06–11.92)	2.57 (1.46–4.53)
Smoking status	Yes	1	510.93	1.96 (0.00–5.87)	2	5184.91	0.39 (0.00–0.96)	5.07 (0.46–55.96)
	No	16	1801.79	8.88 (5.00–13.32)	74	18,787.67	3.94 (3.09–4.84)	2.25 (1.31–3.87)
	Ex-Smoking	0	121.56	0.00 (0.00–0.00)	1	1234.79	0.81 (0.00–2.43)	0.00 (0.00–nan)
Frequency of alcohol consumption (per week)	0	12	1588.7	7.55 (3.78–11.96)	65	16,827.11	3.86 (2.97–4.81)	1.96 (1.06–3.62)
	1–2	4	773	5.17 (1.29–10.35)	10	7581.34	1.32 (0.53–2.24)	3.92 (1.23–12.51)
	≥ 3	1	255.19	3.92 (0.00–11.76)	3	2379.28	1.26 (0.00–2.94)	3.11 (0.32–29.88)
BMI (kg/m^2^)	< 18.5	0	55.51	0.00 (0.00–0.00)	0	408.84	0.00 (0.00–0.00)	nan (nan–nan)
	18.5 to < 25	6	1545.99	3.88 (1.29–7.12)	27	16,339.55	1.65 (1.04–2.33)	2.35 (0.97–5.69)
	≥ 25	12	1073.82	11.18 (5.59–17.69)	51	10,818.96	4.71 (3.51–6.01)	2.37 (1.26–4.45)
Total cholesterol (mg/dL)	< 200	7	1428.03	4.90 (1.40–9.10)	26	14,580.68	1.78 (1.17–2.47)	2.75 (1.19–6.33)
	≥ 200	11	1256.01	8.76 (3.98–14.33)	52	12,994.04	4.00 (2.92–5.16)	2.19 (1.14–4.19)
Income	Low	5	771.13	6.48 (1.30–12.97)	24	8011.64	3.00 (1.87–4.24)	2.16 (0.83–5.67)
	High	13	1912.9	6.80 (3.14–10.98)	54	19,571.04	2.76 (2.04–3.53)	2.46 (1.34–4.51)

*IR* incidence rate, *IRR* incidence rate ratio, *CI* confidence interval, *THA* total hip arthroplasty.

In analyses stratified by lifestyle factors, the incidence of THA was significantly higher in the patient group than in the control group among non-smokers (IRR, 2.25; 95% CI, 1.31–3.87). A significantly increased risk was also observed among non-drinkers (IRR, 1.96; 95% CI, 1.06–3.62) and among individuals who consumed alcohol 1–2 times per week (IRR, 3.92; 95% CI, 1.23–12.51).

In analyses stratified by metabolic indicators, the risk of THA was significantly increased among patients with a BMI ≥ 25 kg/m^2^ (IRR, 2.37; 95% CI, 1.26–4.45). A significantly elevated risk was observed in both subgroups with total cholesterol levels < 200 mg/dL (IRR, 2.75; 95% CI, 1.19–6.33) and ≥ 200 mg/dL (IRR, 2.19; 95% CI, 1.14–4.19). In addition, the risk of THA in the patient group was significantly higher than that in the control group among individuals with higher income levels (IRR, 2.46; 95% CI, 1.34–4.51) (Table 2).

In contrast, crude incidence rate analyses for TKA showed no significant overall increase in risk. Although a trend toward increased risk was observed in certain subgroups, statistical significance was limited. Detailed crude incidence rates and incidence rate ratios for TKA in the LFS group that included lumbar deformity correction and pelvic fixation are presented in Table S3.

Additional analyses assessed THA and TKA incidence in the LFS group without deformity correction or pelvic fixation. THA showed a nonsignificant trend toward increased risk, with higher incidence in some subgroups (e.g., older women). In contrast, TKA incidence was significantly higher in the patient group overall and among individuals aged ≥ 60 years and women. Detailed results are presented in Table S4.

### Multivariate cox regression analysis

In multivariable Cox analyses, LFS with deformity correction and pelvic fixation was significantly associated with increased THA risk (aHR, 2.26; 95% CI, 1.33–3.82), consistent across adjusted models. The association was evident among individuals aged ≥ 60 years (aHR, 2.46; 95% CI, 1.42–4.24) and women (aHR, 2.19; 95% CI, 1.25–3.83), and remained significant among non-smokers, non-drinkers, individuals with BMI ≥ 25 kg/m^2^, and those with higher total cholesterol levels and income (Table 4). In contrast, no significant association was observed for TKA in the overall cohort (Table S5).

**Table 4:** Multivariate Cox proportional hazards regression analysis of the association between LFS and the risk of THA.

		HR (95% CI)			
		Unadjusted	Model 1 ^a^	Model 2^b^	Model 3 ^c^
All		2.37 (1.42–3.95)	2.35 (1.41–3.92)	2.25 (1.33–3.81)	2.26 (1.33–3.82)
Age (years)	< 60	1.13 (0.14–8.96)	1.15 (0.15–9.07)	0.86 (0.10–7.30)	0.63 (0.07–5.92)
	≥ 60	2.55 (1.50–4.33)	2.53 (1.49–4.30)	2.44 (1.41–4.20)	2.46 (1.42–4.24)
Sex	Male	2.28 (0.49–10.54)	2.50 (0.54–11.62)	2.60 (0.56–12.17)	3.01 (0.64–14.01)
	Female	2.39 (1.39–4.11)	2.33 (1.35–4.02)	2.22 (1.27–3.88)	2.19 (1.25–3.83)
Smoking status	Yes	5.21 (0.47–57.51)	8.76 (0.69–110.88)	10.32 (0.77–138.07)	18.08 (1.06–309.13)
	No	2.25 (1.31–3.86)	2.16 (1.26–3.71)	2.18 (1.27–3.74)	2.17 (1.27–3.73)
Frequency of alcohol consumption (per week)	0	1.95 (1.05–3.60)	1.90 (1.03–3.53)	1.93 (1.04–3.58)	1.93 (1.04–3.58)
	1–2	3.92 (1.23–12.50)	2.98 (0.91–9.76)	3.05 (0.93–9.96)	2.84 (0.84–9.55)
	≥ 3	3.10 (0.32–29.81)	3.82 (0.37–39.02)	3.84 (0.29–51.36)	25.82 (0.94–709.09)
Total cholesterol (mg/dL)	< 200	2.74 (1.19–6.30)	2.80 (1.21–6.45)	2.47 (1.02–6.01)	2.51 (1.03–6.12)
	≥ 200	2.19 (1.14–4.20)	2.09 (1.09–4.01)	2.12 (1.10–4.08)	2.13 (1.11–4.09)
BMI (kg/m^2^)	18.5 to < 25	2.34 (0.97–5.67)	2.35 (0.97–5.69)	1.92 (0.74–4.99)	1.90 (0.73–4.93)
	≥ 25	2.37 (1.26–4.44)	2.38 (1.27–4.46)	2.37 (1.26–4.45)	2.41 (1.28–4.53)
Income	Low	2.15 (0.82–5.63)	2.42 (0.92–6.36)	2.20 (0.76–6.38)	2.02 (0.69–5.86)
	High	2.47 (1.35–4.52)	2.37 (1.29–4.34)	2.36 (1.29–4.33)	2.36 (1.29–4.33)

*LFS* lumbar fusion surgery, *THA* total hip arthroplasty, *HR* hazard ratio, *CI* confidence interval.

^a^Adjusted for age and sex 2.

^b^Adjusted for age, sex, smoking status, BMI 6.

^c^Adjusted for age, sex, smoking status, BMI, total cholesterol, income 8.

Subgroups with insufficient events (ex-smokers and BMI < 18.5 kg/m^2^) were excluded from the analysis (NA).

In analyses of the LFS group without lumbar deformity correction or pelvic fixation, increased risks of THA and TKA were observed in certain subgroups; however, the overall pattern of associations and statistical significance were more limited compared with LFS with deformity correction group. Detailed aHRs and subgroup analysis results for the risks of THA and TKA are presented in Table S6.

### Kaplan–meier survival and forest plot analyses

**Fig. 3:**
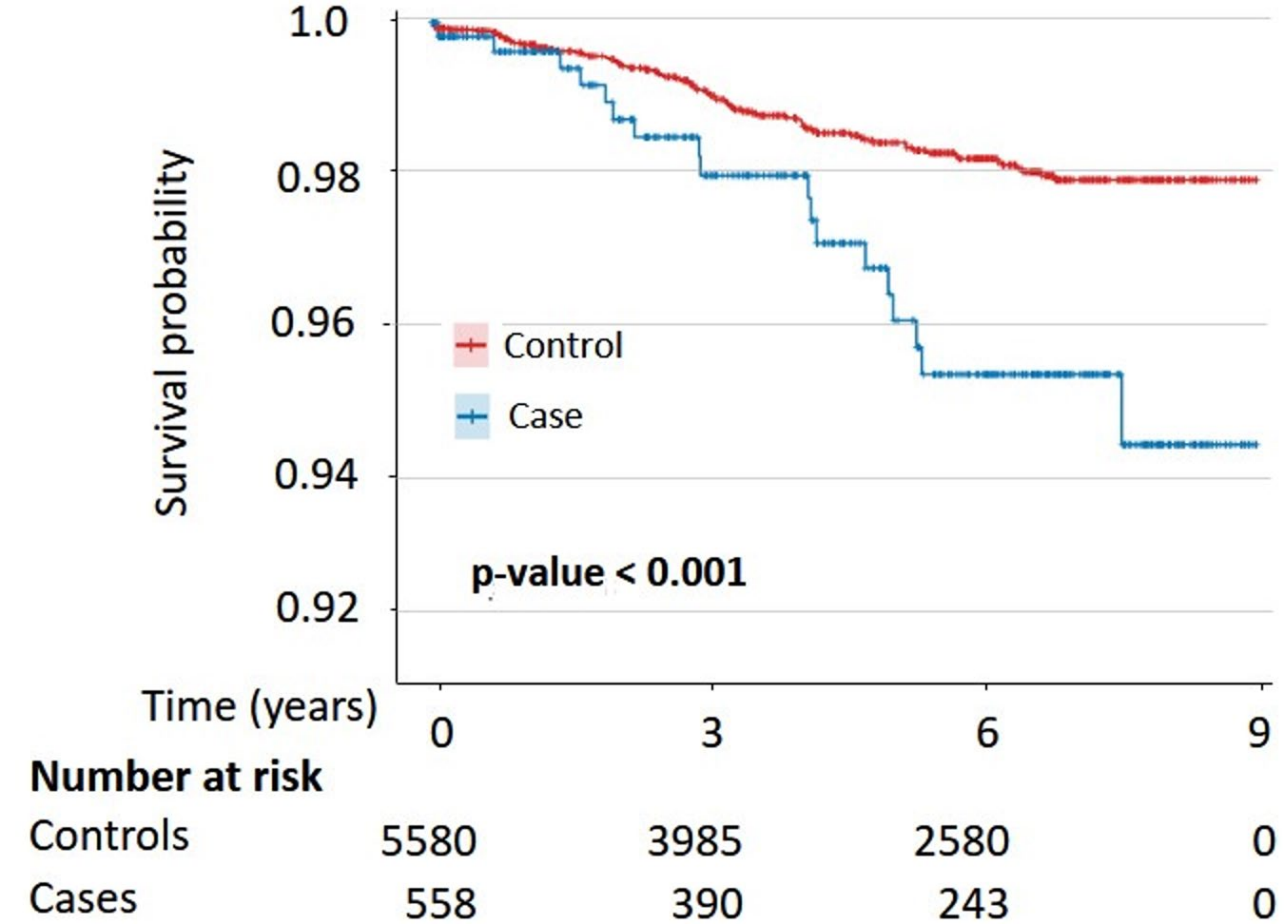
Kaplan–Meier curves showing the cumulative survival probability of primary total hip arthroplasty (THA) after lumbar fusion surgery with deformity correction.

Kaplan–Meier analyses showed a significantly higher cumulative incidence of THA in the LFS group with deformity correction and pelvic fixation than in controls, with a more rapid decline in survival probability and a significant log-rank test result (Fig. 3). In contrast, TKA curves were similar between groups with no significant difference by log-rank test (Figure S1).

**Fig. 4:**
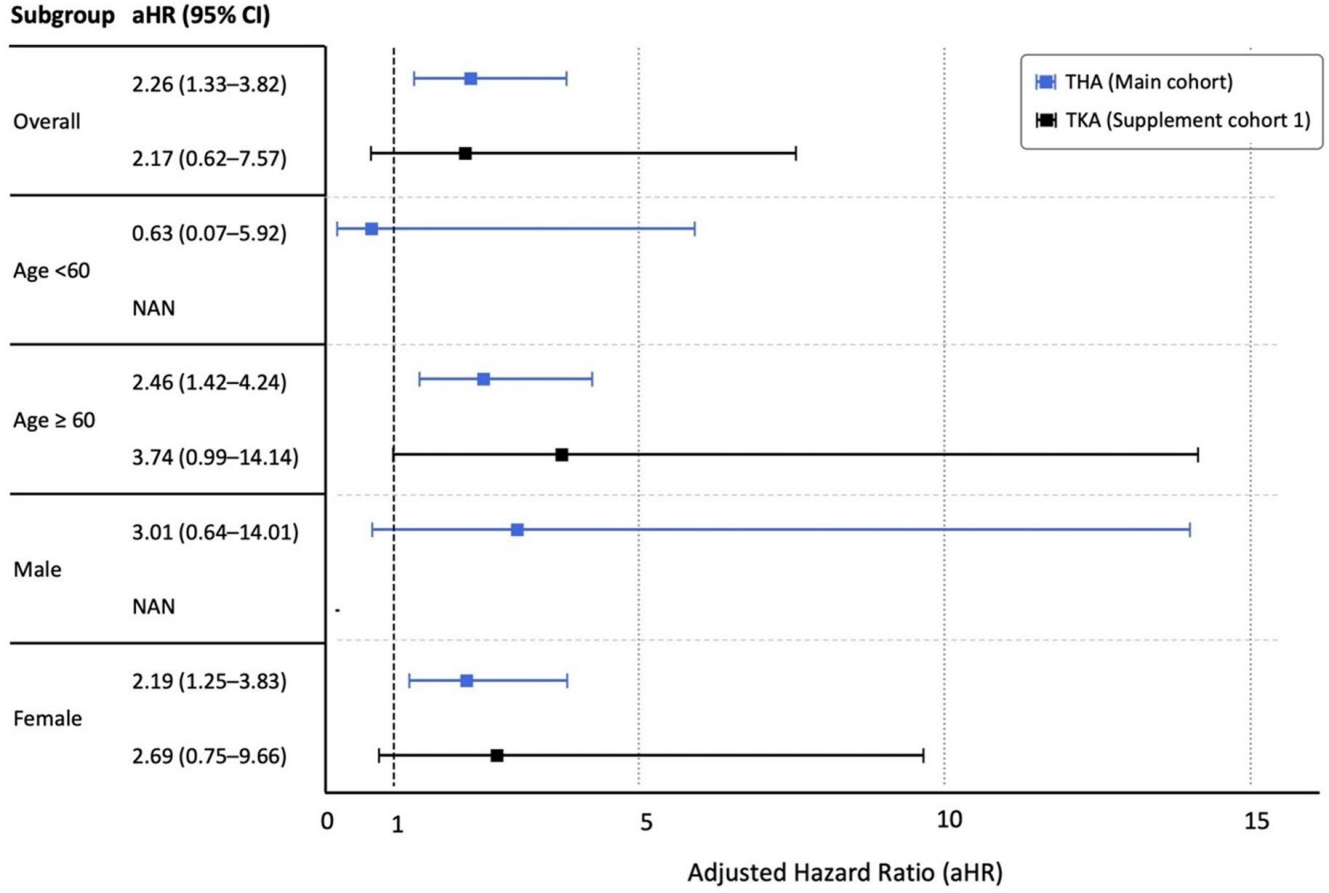
Forest plot of adjusted hazard ratios for primary arthroplasty in patients who underwent lumbar fusion surgery with deformity correction compared to matched controls using a multivariable Cox proportional hazards model. THA, total hip arthroplasty; TKA, total knee arthroplasty. aHR: adjusted Hazard Ratio; CI: Confidence Interval. *Note* Adjusted for age, sex, smoking status, alcohol consumption, body mass index (BMI), total cholesterol and income.

These findings were consistent in forest plot analyses based on multivariable Cox models (Fig. 4). For THA, the overall aHR was 2.26 (95% CI, 1.33–3.82), with particularly elevated risks among patients aged ≥ 60 years (aHR, 2.46; 95% CI, 1.42–4.24) and women (aHR, 2.19; 95% CI, 1.25–3.83). In contrast, the overall aHR for TKA was 2.17 (95% CI, 0.62–7.57) and was not statistically significant, with most age- and sex-stratified confidence intervals including 1.

The cumulative risks of THA and TKA were also evaluated in the LFS group without lumbar deformity correction or pelvic fixation. Although the overall trends were partially like those observed in the LFS group that included deformity correction and pelvic fixation, the statistical significance and consistency of the associations were relatively limited. Detailed Kaplan–Meier curves and forest plot results for this group are presented in Figures S2 and S3.

## Discussion

### Overall study findings

This large-scale population-based cohort study confirmed that patients who underwent lumbar fusion surgery (LFS), including deformity correction, have a significantly increased risk of subsequently undergoing primary total hip arthroplasty (THA).

In the multivariate analysis adjusted for sex, age, and clinical characteristics, the incidence rate ratio (IRR) for THA in LFS patients was 2.37 (95% CI 1.42–3.96), and the adjusted hazard ratio (aHR) was 2.26 (95% CI 1.33–3.82).

These findings suggest that lumbar fusion surgery is associated with degenerative changes in major lower extremity joints and the subsequent progression to total joint arthroplasty.

### Pathophysiology

The increased risk of THA following LFS is attributed to the biomechanical interdependence of the spinopelvic–hip complex. Normally, when transitioning from standing to sitting, the pelvis rotates posteriorly (decreasing the sacral slope) to secure the functional anterior space of the acetabulum and minimize impingement during hip flexion^12,13^. However, LFS restricts or eliminates the key compensatory mechanism of posterior pelvic tilt, and in some cases, even reverses it^14^. Consequently, the risk of femoroacetabular impingement increases, inducing excessive stress on the hip cartilage and surrounding soft tissues^15^.

Specifically, sacropelvic fixation associated with deformity correction substantially reduces spinal and pelvic mobility, limiting compensatory pelvic motion and thereby increasing mechanical loading on the hip joint during daily activities, which may accelerate degenerative changes^16–18^. Various motion analysis and finite element studies have also reported that sacropelvic fixation significantly increases joint surface stress and range of motion in the hip^19–21^.

The loss of such spinopelvic compensatory mechanisms has been widely established as the mechanistic basis for degenerative changes leading to THA, and as shown in our results, it is estimated to accelerate hip degeneration and facilitate progression to surgery.

Furthermore, the atrophy and weakness of paraspinal muscles, such as the multifidus and erector spinae, as well as the gluteal muscles following LFS, can further impair the dynamic stability of the lower extremity joints^1,22^. The use of a posterior approach requires extensive dissection and retraction of deep stabilizing muscles, which can cause muscle, nerve, and vascular damage, thereby concentrating additional mechanical loads on the hip joint during postural maintenance and gait^4^. Over time, these changes accelerate cartilage damage and joint surface wear, increasing the risk of long-term hip degeneration^6^. In summary, LFS establishes a pathophysiological foundation that eliminates key hip compensatory mechanisms, concentrates mechanical loads, and accelerates long-term degeneration. These pathophysiological pathways support our findings of a significantly increased risk of THA in LFS patients and suggest that the impact may be even more pronounced when the fusion extends to the pelvis.

### Comparison with previous studies: THA vs TKA

In this study, the risk of THA following LFS increased more significantly than TKA. Multivariate Cox regression yielded an adjusted hazard ratio of over 2.0 for THA, while TKA showed no significance. This stems from the direct mechanical linkage of the spinopelvic-hip axis. Since the hip is anatomically joined to the pelvis, LFS-induced pelvic rigidity concentrates mechanical loads on hip cartilage^23–26^. One study reported accelerated joint space narrowing in the hip after lumbosacral fusion, especially in long-segment fusions^27^. While LFS is known to increase complications after THA^28^. However, most of these were conducted on patients who had already undergone THA, and it has not been clearly established whether LFS itself increases the primary risk of THA by inducing hip degeneration.

In contrast, TKA-related studies remain limited to small reports suggesting only potential biomechanical shifts^29,30^. Our analysis showed limited TKA risk, suggesting the knee is less vulnerable to sagittal alignment changes due to lower anatomical dependence on spinopelvic mechanisms^23,24^. These results align with the hypothesis of preferential load concentration on the hip after LFS^25–28^.

Moreover, in the analysis of the lumbar fusion group without sacropelvic fixation, the increased risks for THA and TKA were less prominent or statistically non-significant. This highlights that the risk of hip degeneration may be determined by the extent of surgery and the level of fixation, particularly sacropelvic involvement, rather than LFS itself^27–29^. Consequently, hip degeneration risk appears to be a heterogeneous outcome that varies depending on the mechanical characteristics and technical components of the LFS.

However, caution is required in generalizing clinical conclusions from the secondary analyses due to limitations such as reduced sample sizes, shorter follow-up periods, and the absence of radiographic parameters. Nevertheless, this study directly compared THA and TKA risks within the same cohort and addressed a gap in the literature by providing epidemiological data to support the hypothesis that the hip is more vulnerable to the mechanical impacts of LFS than the knee.

### Age

The increased risk of THA following LFS with deformity correction and sacropelvic fixation was significantly observed only in patients aged 60 years and older, with no marked increase in those under 60 years.

This suggests that cumulative degenerative changes, sarcopenia, and gait instability in older age may interact with reduced spinal flexibility after LFS, accelerating long-term hip joint damage. Population-based studies consistently report an increased prevalence of hip osteoarthritis and the need for THA in older populations^31^.

### Sex

In the LFS group with deformity correction and sacropelvic fixation, the risk of THA increased significantly in females, while males showed only a non-significant increasing trend.

The supplementary TKA analysis for the group without deformity correction similarly showed a prominent increase in incidence only among females. Despite a higher prevalence of osteoarthritis, females often undergo THA/TKA later than males due to different surgical preferences^32,33^. The marked risk in females suggests that post-LFS mechanical stress exceeded a critical threshold, emphasizing the need to consider sex-specific factors in surgical decision-making.

### BMI

In the LFS group with deformity correction and sacropelvic fixation, THA risk increased significantly in patients with BMI ≥ 25 kg/m^2^, with a similar increasing trend in the normal weight group (18.5–25 kg/m^2^).

Obesity increases compressive forces on the hip and impairs cartilage metabolism through chronic inflammation^34^. These factors add mechanical stress to the already limited spinopelvic compensation after LFS, accelerating hip degeneration.

Similar trends were observed in the group without deformity correction, though statistical significance was limited. Consistent with studies linking weight gain to higher THA/TKA risk, these findings highlight the importance of weight management in LFS patients^35^.

### Income

In the LFS group with deformity correction and sacropelvic fixation, the increased THA risk was more pronounced in the high-income subgroup. This suggests that post-LFS hip degeneration is influenced by socioeconomic factors, such as healthcare access and activity expectations, alongside biological factors. Previous studies have reported healthcare disparities where lower-income groups show lower or delayed arthroplasty rates despite surgical indications^36^.

The clearer manifestation of THA risk in high-income patients likely stems from their greater willingness to seek surgical solutions and superior healthcare access. Therefore, shared decision-making considering socioeconomic factors is essential when discussing THA/TKA risk after LFS.

### Implications

Our findings indicate that LFS patients, particularly the elderly and females, face a significantly increased long-term risk of degenerative changes in the hip and knee, leading to subsequent arthroplasty. Furthermore, higher BMI and income levels were significantly associated with increased incidence and risk of THA. This emphasizes the need for clinicians to adopt a comprehensive approach, considering systemic biomechanical changes across the spinopelvic-hip axis rather than focusing solely on the spine. Therefore, regular monitoring of hip pain and functional changes in elderly, female, and obese patients can facilitate early detection and timely intervention.

### Study limitations

Despite the strengths of a large nationwide cohort, this study has several limitations.

First, although a 3-year washout period was applied, some patients who already required THA may have undergone LFS first, potentially overestimating the observed THA risk.

Second, because this analysis was based on claims data, important surgical details—including fusion levels, the number of fused segments, and postoperative spinopelvic changes—were unavailable.

Third, we could not evaluate clinical information such as radiologic severity of hip degeneration, muscle strength, or functional recovery.

Fourth, potential confounding related to the control group selection cannot be entirely excluded. LFS patients likely had more severe preoperative spinal deformity, sagittal imbalance, or joint degeneration compared to non-surgical controls. Thus, the increased risk may reflect preexisting degenerative states rather than the effects of fusion alone, requiring cautious interpretation.

Nevertheless, we minimized confounding through large-scale long-term follow-up and rigorous matching; future studies incorporating radiographic and clinical assessments are warranted.

## Conclusion

Lumbar fusion with deformity correction and sacropelvic fixation was associated with a significantly increased long-term risk of primary THA, supporting the biomechanical vulnerability of the hip to spinopelvic alignment changes after surgery. These findings highlight the importance of monitoring hip health and considering spinopelvic–hip biomechanics when planning and managing lumbar fusion procedures.

## Supplementary Information

Below is the link to the electronic supplementary material.

Supplementary Material 1

## Data Availability

The dataset used in this study was obtained from the Korean National Health Insurance Service (NHIS) and is not publicly available due to data privacy regulations. Data access requires approval from the NHIS and cannot be shared publicly. Requests for data access should be directed to the NHIS (https://nhiss.nhis.or.kr) or to the corresponding author, Dr. Hohyun Jung (hhjung@sungshin.ac.kr).
