# Supplementary material for: Risk of total hip arthroplasty following lumbar fusion surgery in a nationwide cohort study
Source: Sci Rep. 2026 Jan 18;16:5670. doi: 10.1038/s41598-026-35894-8 (PMC12891474; doi:10.1038/s41598-026-35894-8)
Supplement: Supplementary file 1 — Supplementary Material 1 [file 41598_2026_35894_MOESM1_ESM.docx]

**Supplementary material**

**Table S1.** International Classification of Diseases, 10th Revision (ICD-10) or Korean Standard Classification of Diseases (KCD) code used for exclusion criteria in study cohort selection

| Disease | ICD-10 / KCD code |
| --- | --- |
| Fracture | |
| Pelvic fracture | S32.0, S32.1, S32.2, S32.3, S32.4, S32.5, S32.6, S32.7, S32.8 |
| Hip fracture | S72.0, S72.1, S72.2, S32.4 |
| Non-traumatic hip joint pathologies | |
| Femoral Head Osteonecrosis (AVN) | M87.0, M87.1, M87.2, M87.3 |
| Infectious arthritis/Osteomyelitis | M00.0, M00.8, M00.9, M60.x, M86.x |
| Advanced Hip Osteoarthritis | M16.0, M16.1, M16.2, M16.3, M16.4, M16.5, M16.6, M16.7, M16.9 |
| Inflammatory Arthropathies | |
| Rheumatoid arthritis | M05.x, M06.x |
| Ankylosing spondylitis | M45.x |
| Psoriatic arthritis | M07.0, M07.1, M07.2, M07.3, L40.5 |
| Other spondyloarthropathies | M46.x |
| Systemic lupus erythematosus (optional) | M32.x |
| Tumor involving hip/pelvis | |
| Primary malignant bone tumor | C40.2 (femur), C41.x (pelvis/spine) |
| Metastatic bone tumor | C79.5 |
| Benign tumor (optional) | D16.x |
| Neuromuscular disorders | |
| Cerebral palsy | G80.x |
| Poliomyelitis sequelae | B91 |
| Muscular dystrophy | G71.x |
| Parkinson disease (advanced only) | G20 |
| Paralytic sequelae of stroke | I69.3, I69.4 |

**Table S2.** Baseline characteristics of patients with lumbar fusion without deformity correction and matched controls. BMI, body mass index; FBS, fasting blood sugar.

|  | | **Case group**  **(n =526) (%)** | **Control group**  **(n = 5,260) (%)** | **Standardized difference** | ***p*-value** |
| --- | --- | --- | --- | --- | --- |
| Age (years) | 20–29 | 5 (0.95%) | 50 (0.95%) | 0.0 | 1 |
|  | 30–39 | 25 (4.75%) | 250 (4.75%) |  |  |
|  | 40–49 | 99 (18.82%) | 990 (18.82%) |  |  |
|  | 50–59 | 156 (29.66%) | 1560 (29.66%) |  |  |
|  | 60–69 | 163 (30.99%) | 1630 (30.99%) |  |  |
|  | ≥70 | 78 (14.83%) | 780 (14.83%) |  |  |
| Sex | Male | 230 (43.73%) | 2300 (43.73%) | 0.0 | 1 |
|  | Female | 296 (56.27%) | 2960 (56.27%) |  |  |
| Smoking status | Yes | 99 (18.82%) | 916 (17.41%) | 0.06 | 0.79 |
|  | No | 355 (67.49%) | 3647 (69.33%) |  |  |
|  | Ex-Smoking | 34 (6.46%) | 349 (6.63%) |  |  |
| Frequency of alcohol consumption (per week) | 0 | 331 (62.93%) | 3373 (64.13%) | 0.05 | 0.84 |
|  | 1–2 | 136 (25.86%) | 1332 (25.32%) |  |  |
|  | ≥3 | 49 (9.32%) | 440 (8.37%) |  |  |
| Weight (kg, mean ± SD) | | 63.20 ± 10.37 | 62.73 ± 10.06 | 0.05 | 0.3 |
| Height (cm, mean ± SD) | | 160.26 ± 8.80 | 160.18 ± 8.86 | 0.01 | 0.85 |
| BMI (kg/m^2^) | <18.5 | 9 (1.71%) | 92 (1.75%) | 0.04 | 0.86 |
|  | 18.5 to <25 | 302 (57.41%) | 3088 (58.71%) |  |  |
|  | ≥25 | 214 (40.68%) | 2075 (39.45%) |  |  |
| Total cholesterol (mg/dL) | <200 | 280 (53.23%) | 2850 (54.18%) | 0.06 | 0.67 |
|  | ≥200 | 246 (46.77%) | 2404 (45.70%) |  |  |
| Systolic Blood Pressure (mmHg) | <120 | 154 (29.28%) | 1554 (29.54%) | 0.04 | 0.93 |
|  | 120 to <140 | 273 (51.90%) | 2751 (52.30%) |  |  |
|  | ≥140 | 99 (18.82%) | 952 (18.10%) |  |  |
| Diastolic Blood Pressure (mmHg) | <80 | 236 (44.87%) | 2384 (45.32%) | 0.04 | 0.94 |
|  | 80 to <90 | 211 (40.11%) | 2109 (40.10%) |  |  |
|  | ≥90 | 79 (15.02%) | 764 (14.52%) |  |  |
| FBS (mg/dL) | <100 | 334 (63.50%) | 3379 (64.24%) | 0.03 | 0.96 |
|  | 100 to <126 | 139 (26.43%) | 1377 (26.18%) |  |  |
|  | ≥126 | 53 (10.08%) | 503 (9.56%) |  |  |
| Income | Low | 151 (28.71%) | 1468 (27.91%) | 0.03 | 0.74 |
|  | High | 375 (71.29%) | 3792 (72.09%) |  |  |

**Table S3.** Crude incidence rates and incidence rate ratios of TKA following lumbar fusion with deformity correction, IR, incidence rate; IRR, incidence rate ratio; CI, confidence interval; LFS, lumbar fusion surgery; TKA, total knee arthroplasty; THA, total hip arthroplasty.

|  | | | **Case cohort**  **(n = 558)** | | | **Reference cohort**  **(n = 5,580)** | | | **IRR**  **(95% CI)** |
| --- | --- | --- | --- | --- | --- | --- | --- | --- | --- |
|  |  |  | **Cases** | **Person-years** | **IR per 1000 person-years (95% CI)** | **Cases** | **Person-years** | **IR per 1000 person-years (95% CI)** |  |
| All |  | | 3 | 2720.65 | 1.10 (0.00–2.57) | 17 | 27714.74 | 0.61 (0.32–0.94) | 1.80 (0.53–6.13) |
| Age (years) | | <60 | 0 | 1655.69 | 0.00 (0.00–0.00) | 7 | 16932.05 | 0.41 (0.12–0.77) | 0.00 (0.00–nan) |
|  |  | ≥60 | 3 | 1064.96 | 2.82 (0.00–6.57) | 10 | 10782.7 | 0.93 (0.37–1.58) | 3.04 (0.84–11.04) |
| Sex | | Male | 0 | 1268.81 | 0.00 (0.00–0.00) | 4 | 12908.26 | 0.31 (0.08–0.62) | 0.00 (0.00–nan) |
|  |  | Female | 3 | 1451.85 | 2.07 (0.00–4.82) | 13 | 14806.48 | 0.88 (0.41–1.42) | 2.35 (0.67–8.26) |
| Sex & Age (years) | | Male, <60 | 0 | 854.63 | 0.00 (0.00–0.00) | 1 | 8627.68 | 0.12 (0.00–0.35) | 0.00 (0.00–nan) |
|  |  | Male, ≥60 | 0 | 414.18 | 0.00 (0.00–0.00) | 3 | 4280.59 | 0.70 (0.00–1.64) | 0.00 (0.00–nan) |
|  |  | Female, <60 | 0 | 801.06 | 0.00 (0.00–0.00) | 6 | 8304.37 | 0.72 (0.24–1.32) | 0.00 (0.00–nan) |
|  |  | Female, ≥60 | 3 | 650.78 | 4.61 (0.00–10.76) | 7 | 6502.11 | 1.08 (0.31–2.00) | 4.28 (1.11–16.56) |
| Smoking status | | Yes | 0 | 515.58 | 0.00 (0.00–0.00) | 0 | 5186.39 | 0.00 (0.00–0.00) | nan (nan–nan) |
|  |  | No | 3 | 1832.84 | 1.64 (0.00–3.82) | 12 | 18934.57 | 0.63 (0.32–1.00) | 2.58 (0.73–9.15) |
|  |  | Ex-Smoking | 0 | 121.56 | 0.00 (0.00–0.00) | 2 | 1230.45 | 1.63 (0.00–4.06) | 0.00 (0.00–nan) |
| Frequency of alcohol consumption (per week) | | 0 | 3 | 1614.2 | 1.86 (0.00–4.34) | 12 | 16949.77 | 0.71 (0.35–1.12) | 2.63 (0.74–9.30) |
|  |  | 1–2 | 0 | 780.21 | 0.00 (0.00–0.00) | 3 | 7595.87 | 0.39 (0.00–0.92) | 0.00 (0.00–nan) |
|  |  | ≥3 | 0 | 258.19 | 0.00 (0.00–0.00) | 1 | 2378.98 | 0.42 (0.00–1.26) | 0.00 (0.00–nan) |
| BMI (kg/m^2^) | | <18.5 | 0 | 55.51 | 0.00 (0.00–0.00) | 0 | 408.84 | 0.00 (0.00–0.00) | nan (nan–nan) |
|  |  | 18.5 to <25 | 3 | 1552.52 | 1.93 (0.00–4.51) | 11 | 16374.28 | 0.67 (0.31–1.10) | 2.88 (0.80–10.31) |
|  |  | ≥25 | 0 | 1103.92 | 0.00 (0.00–0.00) | 6 | 10916.3 | 0.55 (0.18–1.01) | 0.00 (0.00–nan) |
| Total cholesterol (mg/dL) | | <200 | 1 | 1442.02 | 0.69 (0.00–2.08) | 6 | 14615.65 | 0.41 (0.14–0.75) | 1.69 (0.20–14.03) |
|  |  | ≥200 | 2 | 1278.64 | 1.56 (0.00–3.91) | 11 | 13091.14 | 0.84 (0.38–1.37) | 1.86 (0.41–8.40) |
| Income | | Low | 1 | 775.47 | 1.29 (0.00–3.87) | 5 | 8051.52 | 0.62 (0.12–1.24) | 2.08 (0.24–17.77) |
|  |  | High | 2 | 1945.18 | 1.03 (0.00–2.57) | 12 | 19663.22 | 0.61 (0.31–0.97) | 1.68 (0.38–7.53) |

**Table S4.** Crude incidence rates and incidence rate ratios of TKA and THA following lumbar fusion without deformity correction, IR, incidence rate; IRR, incidence rate ratio; CI, confidence interval; LFS, lumbar fusion surgery; TKA, total knee arthroplasty; THA, total hip arthroplasty.

(A) THA

|  | | | **Case cohort**  **(n = 526)** | | | **Reference cohort**  **(n = 5,260)** | | | **IRR**  **(95% CI)** |
| --- | --- | --- | --- | --- | --- | --- | --- | --- | --- |
|  |  |  | **Cases** | **Person-years** | **IR per 1000 person-years (95% CI)** | **Cases** | **Person-years** | **IR per 1000 person-years (95% CI)** |  |
| All |  | | 3 | 2628.92 | 1.14 (0.00–2.66) | 11 | 26631.58 | 0.41 (0.19–0.68) | 2.76 (0.77–9.90) |
| Age (years) | | <60 | 0 | 1619.05 | 0.00 (0.00–0.00) | 2 | 16503 | 0.12 (0.00–0.30) | 0.00 (0.00–nan) |
|  |  | ≥60 | 3 | 1009.87 | 2.97 (0.00–6.93) | 9 | 10128.58 | 0.89 (0.39–1.48) | 3.34 (0.91–12.35) |
| Sex | | Male | 0 | 1245.8 | 0.00 (0.00–0.00) | 6 | 12557.44 | 0.48 (0.16–0.88) | 0.00 (0.00–nan) |
|  |  | Female | 3 | 1383.12 | 2.17 (0.00–5.06) | 5 | 14074.15 | 0.36 (0.07–0.71) | 6.11 (1.46–25.55) |
| Sex & Age (years) | | Male, <60 | 0 | 834.68 | 0.00 (0.00–0.00) | 1 | 8400.46 | 0.12 (0.00–0.36) | 0.00 (0.00–nan) |
|  |  | Male, ≥60 | 0 | 411.12 | 0.00 (0.00–0.00) | 5 | 4156.97 | 1.20 (0.24–2.41) | 0.00 (0.00–nan) |
|  |  | Female, <60 | 0 | 784.37 | 0.00 (0.00–0.00) | 1 | 8102.54 | 0.12 (0.00–0.37) | 0.00 (0.00–nan) |
|  |  | Female, ≥60 | 3 | 598.75 | 5.01 (0.00–11.69) | 4 | 5971.61 | 0.67 (0.17–1.34) | 7.48 (1.67–33.42) |
| Smoking status | | Yes | 0 | 503.36 | 0.00 (0.00–0.00) | 1 | 5066.86 | 0.20 (0.00–0.59) | 0.00 (0.00–nan) |
|  |  | No | 3 | 1759 | 1.71 (0.00–3.98) | 7 | 17853.38 | 0.39 (0.11–0.73) | 4.35 (1.12–16.82) |
|  |  | Ex-Smoking | 0 | 115.89 | 0.00 (0.00–0.00) | 3 | 1344.46 | 2.23 (0.00–5.21) | 0.00 (0.00–nan) |
| Frequency of alcohol consumption (per week) | | 0 | 3 | 1549.98 | 1.94 (0.00–4.52) | 6 | 16183.77 | 0.37 (0.12–0.68) | 5.22 (1.31–20.87) |
|  |  | 1–2 | 0 | 759.19 | 0.00 (0.00–0.00) | 1 | 7482.99 | 0.13 (0.00–0.40) | 0.00 (0.00–nan) |
|  |  | ≥3 | 0 | 251.7 | 0.00 (0.00–0.00) | 4 | 2238.07 | 1.79 (0.45–3.57) | 0.00 (0.00–nan) |
| BMI (kg/m^2^) | | <18.5 | 0 | 55.51 | 0.00 (0.00–0.00) | 0 | 494.65 | 0.00 (0.00–0.00) | nan (nan–nan) |
|  |  | 18.5 to <25 | 3 | 1505.28 | 1.99 (0.00–4.65) | 6 | 15861.67 | 0.38 (0.13–0.69) | 5.27 (1.32–21.07) |
|  |  | ≥25 | 0 | 1059.42 | 0.00 (0.00–0.00) | 5 | 10251.85 | 0.49 (0.10–0.98) | 0.00 (0.00–nan) |
| Total cholesterol (mg/dL) | | <200 | 1 | 1385.94 | 0.72 (0.00–2.16) | 2 | 14491.54 | 0.14 (0.00–0.35) | 5.23 (0.47–57.66) |
|  |  | ≥200 | 2 | 1242.98 | 1.61 (0.00–4.02) | 9 | 12101.15 | 0.74 (0.33–1.24) | 2.16 (0.47–10.01) |
| Income | | Low | 1 | 739.02 | 1.35 (0.00–4.06) | 5 | 7523.11 | 0.66 (0.13–1.33) | 2.04 (0.24–17.43) |
|  |  | High | 2 | 1889.91 | 1.06 (0.00–2.65) | 6 | 19108.47 | 0.31 (0.10–0.58) | 3.37 (0.68–16.70) |

(B) TKA

|  | | | **Case cohort**  **(n = 526)** | | | **Reference cohort**  **(n = 5,260)** | | | **IRR**  **(95% CI)** |
| --- | --- | --- | --- | --- | --- | --- | --- | --- | --- |
|  |  |  | **Cases** | **Person-years** | **IR per 1000 person-years (95% CI)** | **Cases** | **Person-years** | **IR per 1000 person-years (95% CI)** |  |
| All |  | | 17 | 2593.19 | 6.56 (3.47–10.03) | 79 | 26460.71 | 2.99 (2.34–3.67) | 2.20 (1.30–3.71) |
| Age (years) | | <60 | 1 | 1618.92 | 0.62 (0.00–1.85) | 13 | 16475.63 | 0.79 (0.36–1.27) | 0.78 (0.10–5.98) |
|  |  | ≥60 | 16 | 974.26 | 16.42 (9.24–24.63) | 66 | 9985.08 | 6.61 (5.11–8.21) | 2.48 (1.44–4.29) |
| Sex | | Male | 2 | 1240.33 | 1.61 (0.00–4.03) | 7 | 12552.22 | 0.56 (0.16–1.04) | 2.89 (0.60–13.92) |
|  |  | Female | 15 | 1352.86 | 11.09 (5.91–17.00) | 72 | 13908.49 | 5.18 (4.03–6.40) | 2.14 (1.23–3.74) |
| Sex & Age (years) | | Male, <60 | 0 | 834.68 | 0.00 (0.00–0.00) | 0 | 8403.7 | 0.00 (0.00–0.00) | nan (nan–nan) |
|  |  | Male, ≥60 | 2 | 405.65 | 4.93 (0.00–12.33) | 7 | 4148.52 | 1.69 (0.48–3.13) | 2.92 (0.61–14.07) |
|  |  | Female, <60 | 1 | 784.24 | 1.28 (0.00–3.83) | 13 | 8071.93 | 1.61 (0.74–2.60) | 0.79 (0.10–6.05) |
|  |  | Female, ≥60 | 14 | 568.62 | 24.62 (12.31–38.69) | 59 | 5836.56 | 10.11 (7.54–12.85) | 2.44 (1.36–4.36) |
| Smoking status | | Yes | 1 | 498.7 | 2.01 (0.00–6.02) | 1 | 5061.87 | 0.20 (0.00–0.59) | 10.15 (0.63–162.27) |
|  |  | No | 15 | 1728.83 | 8.68 (4.63–13.30) | 76 | 17684.6 | 4.30 (3.34–5.32) | 2.02 (1.16–3.51) |
|  |  | Ex-Smoking | 0 | 115.89 | 0.00 (0.00–0.00) | 1 | 1347.47 | 0.74 (0.00–2.23) | 0.00 (0.00–nan) |
| Frequency of alcohol consumption (per week) | | 0 | 11 | 1525.36 | 7.21 (3.28–11.80) | 70 | 16025.05 | 4.37 (3.37–5.43) | 1.65 (0.87–3.12) |
|  |  | 1–2 | 4 | 751.98 | 5.32 (1.33–10.64) | 8 | 7459.53 | 1.07 (0.40–1.88) | 4.96 (1.49–16.47) |
|  |  | ≥3 | 1 | 248.7 | 4.02 (0.00–12.06) | 1 | 2249.38 | 0.44 (0.00–1.33) | 9.04 (0.57–144.60) |
| BMI (kg/m^2^) | | <18.5 | 0 | 55.51 | 0.00 (0.00–0.00) | 0 | 494.65 | 0.00 (0.00–0.00) | nan (nan–nan) |
|  |  | 18.5 to <25 | 6 | 1498.76 | 4.00 (1.33–7.34) | 22 | 15814.37 | 1.39 (0.82–2.02) | 2.88 (1.17–7.10) |
|  |  | ≥25 | 11 | 1030.21 | 10.68 (4.85–17.47) | 57 | 10128.28 | 5.63 (4.25–7.11) | 1.90 (0.99–3.62) |
| Total cholesterol (mg/dL) | | <200 | 7 | 1371.95 | 5.10 (1.46–9.48) | 26 | 14433.88 | 1.80 (1.18–2.49) | 2.83 (1.23–6.53) |
|  |  | ≥200 | 10 | 1221.24 | 8.19 (3.28–13.92) | 53 | 11987.93 | 4.42 (3.25–5.67) | 1.85 (0.94–3.64) |
| Income | | Low | 4 | 735.56 | 5.44 (1.36–10.88) | 17 | 7484.82 | 2.27 (1.20–3.47) | 2.39 (0.81–7.12) |
|  |  | High | 13 | 1857.63 | 7.00 (3.23–11.30) | 62 | 18975.89 | 3.27 (2.48–4.11) | 2.14 (1.18–3.89) |

**Table S5.** Multivariate Cox proportional hazards regression analysis of the association between lumbar fusion surgery with deformity correction and the risk of TKA. TKA, total knee arthroplasty; HR, hazard ratio; CI, confidence interval.

|  | | **HR (95% CI)** | | | |
| --- | --- | --- | --- | --- | --- |
|  |  | **Unadjusted** | **Model 1 ^a^** | **Model 2^b^** | **Model 3 ^c^** |
| All | | 1.79 (0.53–6.11) | 1.78 (0.52–6.06) | 2.19 (0.63–7.62) | 2.17 (0.62–7.57) |
| Age (years) | <60 | NA | NA | NA | NA |
|  | ≥60 | 3.03 (0.83–10.99) | 3.01 (0.83–10.93) | 3.68 (0.97–13.87) | 3.74 (0.99–14.14) |
| Sex | Male | NA | NA | NA | NA |
|  | Female | 2.34 (0.67–8.21) | 2.31 (0.66–8.12) | 2.74 (0.76–9.82) | 2.69 (0.75–9.66) |
| Smoking status | Yes | NA | NA | NA | NA |
|  | No | 2.57 (0.73–9.12) | 2.50 (0.71–8.88) | 2.50 (0.71–8.87) | 2.48 (0.70–8.79) |
|  | Ex-Smoking | NA | NA | NA | NA |
| Frequency of alcohol consumption (per week) | 0 | 2.62 (0.74–9.27) | 2.58 (0.73–9.16) | 3.20 (0.88–11.69) | 3.14 (0.86–11.50) |
|  | 1–2 | NA | NA | NA | NA |
|  | ≥3 | NA | NA | NA | NA |
| Total cholesterol (mg/dL) | <200 | 1.67 (0.20–13.91) | 1.73 (0.21–14.43) | 1.77 (0.21–14.75) | 1.81 (0.22–15.11) |
|  | ≥200 | 1.84 (0.41–8.31) | 1.78 (0.39–8.06) | 2.40 (0.51–11.33) | 2.41 (0.51–11.39) |
| Income | Low | 2.07 (0.24–17.72) | 2.25 (0.26–19.32) | 2.45 (0.27–22.30) | 2.50 (0.27–22.70) |
|  | High | 1.69 (0.38–7.54) | 1.65 (0.37–7.36) | 2.00 (0.44–9.16) | 1.98 (0.43–9.05) |
| BMI (kg/m^2^) | <18.5 | NA | NA | NA | NA |
|  | 18.5 to <25 | 2.85 (0.80–10.22) | 2.85 (0.80–10.23) | 3.50 (0.94–12.97) | 3.47 (0.94–12.87) |
|  | ≥25 | NA | NA | NA | NA |

^a^ Adjusted for age and sex 2

^b^ Adjusted for age, sex, smoking status, BMI 6

^c^ Adjusted for age, sex, smoking status, BMI, total cholesterol, income 8

**Table S6.** Multivariate Cox proportional hazards regression analysis of the association between lumbar fusion surgery without deformity correction and the risk of THA and TKA. THA, total hip arthroplasty; TKA, total knee arthroplasty; HR, hazard ratio; CI, confidence interval.

(A) THA

|  | | **HR (95% CI)** | | | |
| --- | --- | --- | --- | --- | --- |
|  |  | **Unadjusted** | **Model 1 ^a^** | **Model 2^b^** | **Model 3 ^c^** |
| All | | 2.20 (1.30–3.71) | 2.19 (1.30–3.71) | 2.17 (1.27–3.72) | 2.07 (1.20–3.55) |
| Age (years) | <60 | 0.80 (0.10–6.08) | 0.80 (0.10–6.08) | 0.67 (0.09–5.25) | 0.59 (0.08–4.64) |
|  | ≥60 | 2.50 (1.45–4.32) | 2.50 (1.45–4.32) | 2.47 (1.41–4.34) | 2.38 (1.35–4.18) |
| Sex | Male | 3.26 (0.67–15.79) | 3.26 (0.67–15.79) | 4.13 (0.81–21.01) | 3.77 (0.72–19.68) |
|  | Female | 2.12 (1.21–3.69) | 2.12 (1.21–3.69) | 2.06 (1.16–3.66) | 1.94 (1.09–3.45) |
| Smoking status | Yes | 29.58 (1.44–607.71) | 29.58 (1.44–607.71) | 36.47 (1.27–1047.75) | NA |
|  | No | 1.97 (1.13–3.43) | 1.97 (1.13–3.43) | 2.07 (1.19–3.60) | 1.98 (1.14–3.45) |
|  | Ex-Smoking | NA | NA | NA | NA |
| Frequency of alcohol consumption (per week) | 0 | 1.67 (0.88–3.15) | 1.67 (0.88–3.15) | 1.75 (0.92–3.30) | 1.68 (0.89–3.18) |
|  | 1–2 | 2.75 (0.78–9.70) | 2.75 (0.78–9.70) | 2.67 (0.73–9.82) | 2.30 (0.62–8.61) |
|  | ≥3 | 19.81 (0.84–467.39) | 19.81 (0.84–467.39) | 36.95 (0.65–2108.41) | NA |
| Total cholesterol (mg/dL) | <200 | 3.24 (1.40–7.48) | 3.24 (1.40–7.48) | 2.92 (1.20–7.15) | 2.93 (1.19–7.23) |
|  | ≥200 | 1.69 (0.86–3.32) | 1.69 (0.86–3.32) | 1.79 (0.91–3.52) | 1.75 (0.89–3.45) |
| Income | Low | 3.04 (1.02–9.07) | 3.04 (1.02–9.07) | 2.40 (0.70–8.27) | 2.30 (0.66–8.03) |
|  | High | 2.01 (1.11–3.66) | 2.01 (1.11–3.66) | 2.07 (1.14–3.78) | 2.04 (1.12–3.72) |
| BMI (kg/m^2^) | <18.5 | NA | NA | NA | NA |
|  | 18.5 to <25 | 2.85 (1.16–7.04) | 2.85 (1.16–7.04) | 2.36 (0.89–6.24) | 2.30 (0.87–6.11) |
|  | ≥25 | 2.01 (1.05–3.83) | 2.01 (1.05–3.83) | 2.01 (1.05–3.84) | 1.87 (0.98–3.57) |

^a^ Adjusted for age and sex 2

^b^ Adjusted for age, sex, smoking status, BMI 6

^c^ Adjusted for age, sex, smoking status, BMI, total cholesterol, income 8

(B) TKA

|  | | **HR (95% CI)** | | | |
| --- | --- | --- | --- | --- | --- |
|  |  | **Unadjusted** | **Model 1 ^a^** | **Model 2^b^** | **Model 3 ^c^** |
| All | | 2.74 (0.76–9.82) | 2.72 (0.76–9.76) | 2.74 (0.76–9.86) | 3.08 (0.85–11.21) |
| Age (years) | <60 | NA | NA | NA | NA |
|  | ≥60 | 3.31 (0.90–12.22) | 3.31 (0.90–12.22) | 3.18 (0.86–11.84) | 3.50 (0.94–13.08) |
| Sex | Male | NA | NA | NA | NA |
|  | Female | 6.07 (1.45–25.42) | 6.00 (1.43–25.11) | 5.95 (1.42–24.91) | 5.29 (1.25–22.33) |
| Smoking status | Yes | NA | NA | NA | NA |
|  | No | 4.33 (1.12–16.75) | 4.23 (1.09–16.35) | 4.17 (1.08–16.14) | 3.97 (1.02–15.44) |
|  | Ex-Smoking | NA | NA | NA | NA |
| Frequency of alcohol consumption (per week) | 0 | 5.25 (1.31–21.01) | 5.22 (1.30–20.88) | 5.50 (1.36–22.23) | 4.88 (1.20–19.94) |
|  | 1–2 | NA | NA | NA | NA |
|  | ≥3 | NA | NA | NA | NA |
| Total cholesterol (mg/dL) | <200 | 5.16 (0.47–56.88) | 4.45 (0.40–49.57) | 4.94 (0.41–59.75) | 16.03 (0.90–286.61) |
|  | ≥200 | 2.14 (0.46–9.91) | 2.17 (0.47–10.07) | 2.20 (0.47–10.28) | 2.20 (0.47–10.29) |
| Income | Low | 2.15 (0.25–18.48) | 2.55 (0.29–22.23) | 2.30 (0.26–20.56) | 2.32 (0.26–20.60) |
|  | High | 3.36 (0.68–16.65) | 3.19 (0.64–15.83) | 3.31 (0.66–16.56) | 3.20 (0.64–16.10) |
| BMI (kg/m^2^) | <18.5 | NA | NA | NA | NA |
|  | 18.5 to <25 | 5.19 (1.30–20.75) | 5.07 (1.27–20.29) | 5.16 (1.28–20.81) | 5.42 (1.34–21.94) |
|  | ≥25 | NA | NA | NA | NA |

^a^ Adjusted for age and sex 2

^b^ Adjusted for age, sex, smoking status, BMI 6

^c^ Adjusted for age, sex, smoking status, BMI, total cholesterol, income 8

**Figure S1.** Kaplan–Meier curves showing the cumulative survival probability of primary total knee arthroplasty (TKA) after lumbar fusion surgery without deformity correction


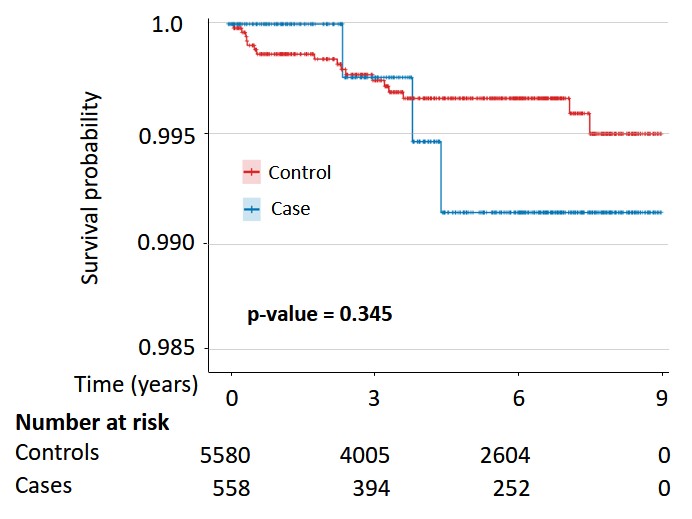


**Figure S2.** Kaplan–Meier curves showing the cumulative survival probability of (1) primary total hip arthroplasty (THA) and (2) primary total knee arthroplasty (TKA) after lumbar fusion surgery without deformity correction

1. Total Hip Arthroplasty (THA).


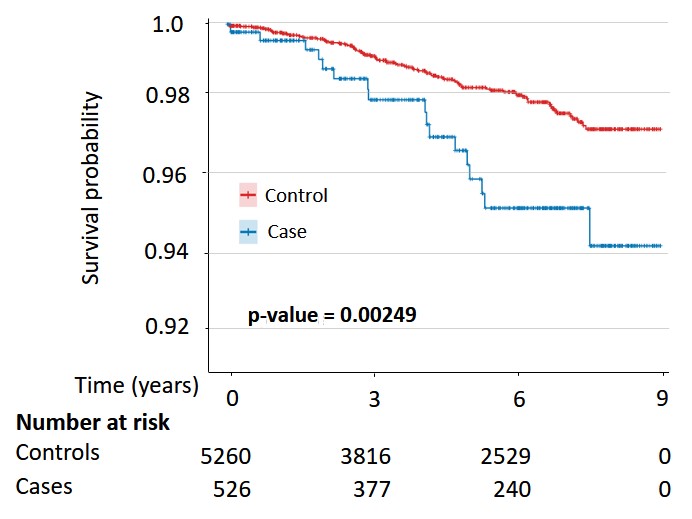


2. Total Knee Arthroplasty (TKA).


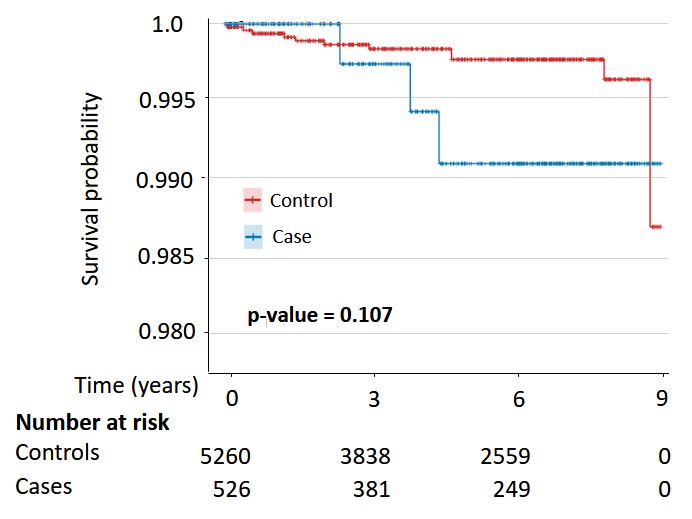


**Figure S3.** Forest plot of adjusted hazard ratios for primary arthroplasty in patients who underwent lumbar fusion surgery without deformity correction compared to matched controls using a multivariable Cox proportional hazards model. THA, total hip arthroplasty; TKA, total knee arthroplasty. aHR : adjusted hazard ratio; CI: confidence interval


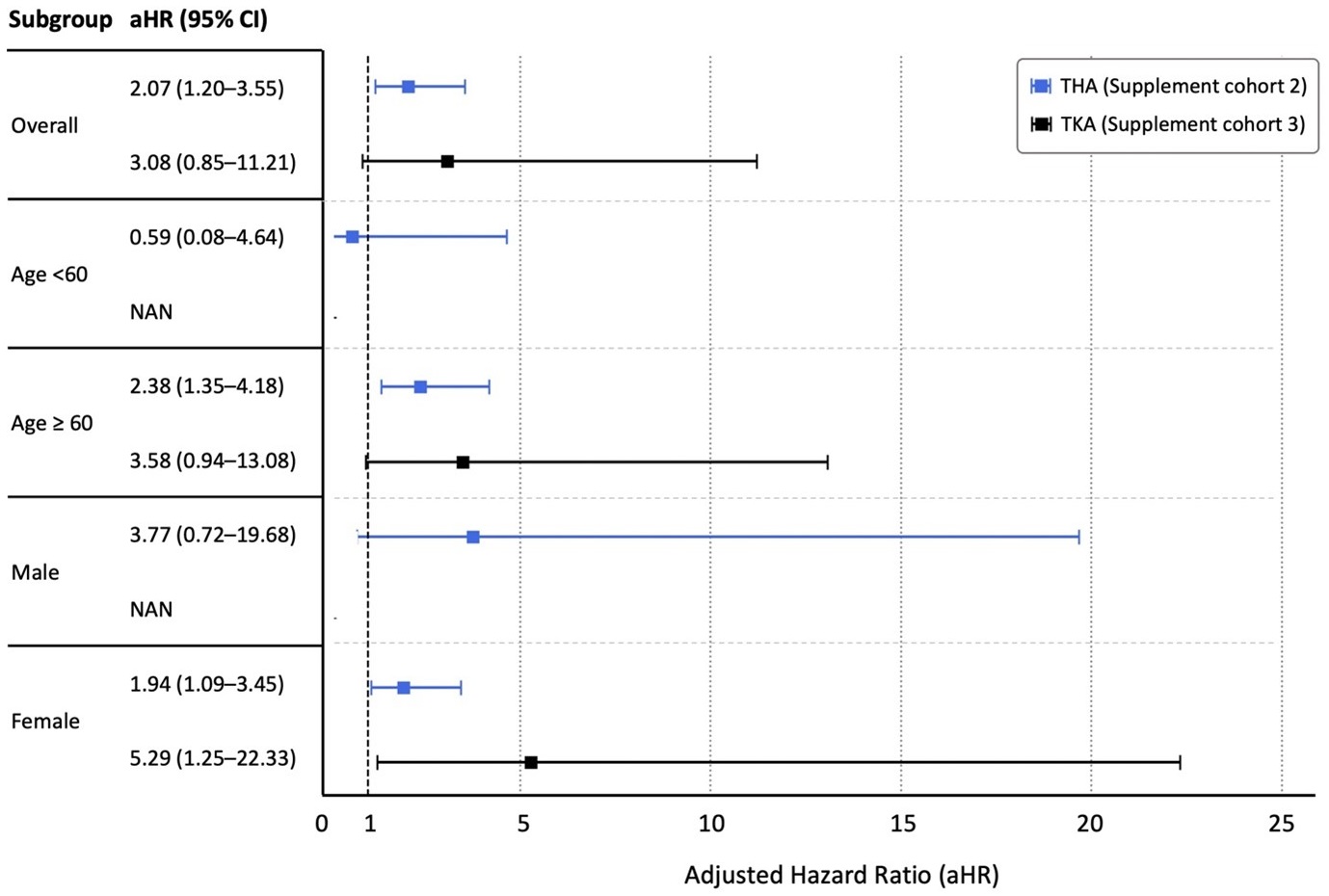


Note. Adjusted for age, sex, smoking status, alcohol consumption, body mass index (BMI), total cholesterol and income.
